# Intradermal Testing Results in Horses Affected by Mild-Moderate and Severe Equine Asthma

**DOI:** 10.3390/ani11072086

**Published:** 2021-07-13

**Authors:** Chiara Maria Lo Feudo, Luca Stucchi, Elena Alberti, Bianca Conturba, Enrica Zucca, Francesco Ferrucci

**Affiliations:** 1 Equine Sports Medicine Laboratory “Franco Tradati”, Department of Veterinary Medicine, Università Degli Studi di Milano, 26900 Lodi, Italy; chiara.lofeudo@unimi.it (C.M.L.F.); elena.alberti@unimi.it (E.A.); enrica.zucca@unimi.it (E.Z.); 2 Veterinary Teaching Hospital, Università Degli Studi di Milano, 26900 Lodi, Italy; luca.stucchi@unimi.it (L.S.); bianca.conturba@unimi.it (B.C.)

**Keywords:** horse, equine asthma, recurrent airway obstruction, skin test, intradermal testing, allergy test

## Abstract

**Simple Summary:**

Equine asthma is a respiratory syndrome sharing several similarities with human asthma and represents the most common cause of chronic coughing in horses. Based on the severity and recurrence of the conditions, it is classified as mild-moderate or severe equine asthma. Although a precise pathogenetic mechanism has not yet been identified, it is generally agreed that environmental allergens behave as triggers of a hypersensitivity response (HR), culminating in asthmatic exacerbations. In human medicine, the skin prick test is considered the gold standard of allergy testing; similarly, in equine medicine, intradermal testing is used to identify hypersensitivities to specific allergens. The present study describes and compares the results of intradermal testing in horses affected by either mild-moderate or severe equine asthma to evaluate the responsiveness of asthmatic horses and to identify the most frequently involved allergens. Type-I HR was shown to play a major role in the pathogenesis of severe equine asthma, while type-IV HR seems to be involved mostly in milder forms. Insects represented the antigens inducing the most frequent and strongest reactions among asthmatic horses, followed by *Dermatophagoides spp.* and dog epithelium; these allergens should therefore be considered for avoidance strategies and the future development of desensitizing allergen-specific immunotherapy.

**Abstract:**

Equine asthma is an inflammatory respiratory disorder, classified as mild-moderate (MEA) and severe (SEA). SEA is characterized by recurrent exacerbations, consisting of dyspnea, coughing and exercise intolerance; MEA causes poor performance, occasional cough and mucus hypersecretion. Although a precise pathogenesis is not completely understood, allergic mechanisms are considered an important pathophysiological feature of equine asthma. In equine medicine, intradermal testing (IDT) is effective in identifying hypersensitivity to specific allergens. However, to date, the studies about IDT in asthmatic horses obtained contradictory results. This study aims to evaluate IDT responses in MEA and SEA horses and to identify the most significant allergens. Thirty-eight asthmatic horses were enrolled and underwent IDT using 50 allergens; reactions were evaluated at 30 min, 4, 24 and 48 h and were assigned a score from 0 to 4. In SEA horses, the most frequent and strongest reactions were observed at 30 min and 4 h, suggesting the involvement of type I hypersensitivity; in MEA horses, also type IV hypersensitivity seemed to play a major role. Insects, *Dermatophagoides spp.* and dog epithelium induced in MEA and SEA horses the most significant hypersensitivity responses and could therefore be considered as the main allergenic antigens in our geographic area.

## 1. Introduction

Equine asthma (EA) is a syndrome including inflammatory, recurrent, and chronic lower airway disorders of adult horses, sharing several similarities with human asthma [1,2]. It is clinically characterized by airflow obstruction, mucus hypersecretion and airway hyperreactivity. Based on the severity and recurrence of the condition, it is classified as mild, moderate or severe equine asthma [1]. Severe equine asthma (SEA) is the most common cause of chronic cough in horses [3], naturally affecting 10–15% of adult horses (typically over seven years of age) living in the northern hemisphere [4] where horses are stabled indoors during most of the year [5]. It is characterized by recurrent and reversible episodes of disease exacerbations, consisting of increased respiratory effort at rest, coughing and exercise intolerance [6]. Mild or moderate equine asthma (MEA), in contrast, can affect horses of any age but is more commonly reported in young horses; poor performance can be the only symptom, generally associated with occasional coughing and mucus hypersecretion, with normal breathing at rest [7].

In asthmatic horses, clinical signs are triggered by the inhalation of antigens present in the stables, such as moulds, spores and mites [8], which may vary according to environmental exposure and geographical area [9]. Seasonal pollens and other antigens found in high levels outdoors in spring and summer represent another group of etiological agents able to cause exacerbations in susceptible horses: this condition is known as summer pasture asthma [1,6]. Although it is generally agreed upon that EA represents a hypersensitivity response (HR) to inhaled antigens, a precise pathogenetic mechanism among susceptible horses has not yet been identified [4]. 

Various studies suggest that IgE-mediated immediate-type allergic reactions are associated with SEA [10,11,12,13]. In fact, the increase in IgEs detected in the bronchoalveolar lavage fluid of the SEA horses is typical of a type I HR [14]. Moreover, histamine release from pulmonary mast cells in response to allergens exposure in vitro is significantly greater in asthmatic horses compared to healthy horses, suggesting the involvement of IgE-mediated reactions leading to increased mast cell degranulation [15]. Mast cell mediators, such as histamine and leukotrienes, induce smooth muscle contraction and may contribute to bronchospasm in SEA [14,16]. Also, a delayed-onset type-IV hypersensitivity seems to be involved in SEA, mediated by Th-2 lymphocytes (CD4+ lymphocyte subgroup) [17,18,19]. Therefore, the identification of specific allergens that induce hypersensitivity reactions could represent an important tool for diagnosis, prognosis and choice of the most appropriate treatment [20,21]. 

For this purpose, in human medicine, the gold standard of allergy tests is considered the skin prick test (SPT), while in atopic horses, intradermal testing (IDT) is the preferred method used to confirm the diagnosis of immediate and delayed hypersensitivity reactions and to select avoidance measures and/or allergen-specific immunotherapy (ASIT) [22,23,24]. After the allergen is inoculated, allergen-specific IgE binds to the high-affinity IgE receptor (FcεRI) on the surface of mast cells, activating a signal transduction cascade, which finally induces mast cell degranulation: consequently, various preformed inflammatory mediators are released and a wheal-and-flare reaction develops [22]. Up to 85% of human asthmatic patients show positive reactions to SPT for common aeroallergens [25]. However, positivity is not specific for asthma nor is it present in all asthma phenotypes: in fact, it identifies atopic status, which increases the probability that a patient with respiratory symptoms has allergic asthma. Thus, it is essential to interpret positive skin test reactions in light of the patient’s history [21]. 

Most healthy patients could have one or more positive reactions to skin tests; however, atopic horses have a significantly greater number of positive reactions [23]. In one study including 86 horses, SEA horses seem to be at least six times as likely to respond to one or more allergens compared to healthy horses, and the mean number of allergens provoking a positive reaction in SEA horses is approximately two to 10 times greater than in healthy horses [26]. Moreover, the mean wheal diameter in SEA horses seems to be significantly greater than in control horses, indicating a greater reactivity [27]: in one study including 40 horses, 98% of the diameters of the wheals in the SEA group were larger than 1 cm, contrasting with 27% in the healthy group [28]. 

However, contrasting results have been obtained from different studies, creating a lack of understanding of IDT as an additional diagnostic tool for equine asthma [10,11,12,24,26,27,28,29,30,31,32]. However, it is difficult to compare findings of different investigations, since IDT and the allergens used in the different studies are not standardized for the horse [24,33]. The present study aims to evaluate IDT reactions and to measure the prevalence of positivity induced by different allergens in horses affected by either SEA or MEA.

## 2. Materials and Methods

### 2.1. Horses

For this retrospective study, clinical records of all horses affected by SEA or MEA for which an IDT was performed between 2008 and 2020 at the Equine Unit of the Veterinary Teaching Hospital of the University of Milan (Italy) were reviewed. To be enrolled, all horses had to be vaccinated against influenza and tetanus and regularly dewormed. Each patient underwent a complete diagnostic evaluation, including clinical history, general physical examination, airway endoscopy and assessment of tracheal mucus, and bronchoalveolar lavage (BAL) cytology [34]. For SEA horses, inclusion criteria were history of exercise intolerance, dyspnea, coughing, normal rectal temperature, absence of alterations at thoracic ultrasound consistent with pneumonia, detection of tracheobronchial mucus grade >2/5 and neutrophils count >25% in BAL cytology [7]. MEA horses were selected on the basis of history of poor performance and occasional cough, eupnea at rest, normal rectal temperature, detection of tracheobronchial mucus grade >2/5 [7] and BAL cytology consisting of neutrophils >5%, and/or mast cells >2% and/or eosinophils >1% [35]. Whenever glucocorticoids were recently administered, they were withdrawn for a minimum of three weeks for oral or topical administration, or eight weeks for injective administration; similarly, antihistamines were withdrawn for a minimum of 10 days because these drugs could cause false negative IDT results [23]. Moreover, none of the horses included in this study had history of dermatological diseases nor had ever received ASIT. 

### 2.2. Intradermal Testing

Fifty allergens, consisting of individual allergens or allergen mixes, were used for IDT, including eight moulds/fungi, five mites, six insects, 20 plants, three animals and eight feedstuffs (Artuvetrin^®^ Skin test, Nextmune, Lelystad, Netherlands; Prick test, Lofarma SPA, Milan, Italy). Mould allergens included *Alternaria alternata*, *Candida albicans*, *Aspergillus fumigatus*, *Cladosporium spp.*, *Helmintosporium sativum*, Mycophytes mix, Trichoepidermophytes mix, and *Penicillium* mix. Mite allergens included *Acarus siro*, *Gliciphagus domesticus*, *Tyrophagus putrescentiae*, *Lepidogliphus destructor* and *Dermatophagoides* mix. Insect allergens included *Culicoides spp.*, *Culex spp.*, *Aedes spp.*, *Tabanus spp.*, *Chrysops spp.* and *Musca domestica*. Plant allergens included *Olea europea*, *Cupressus sempervirens*, *Corylus avellana*, *Cynodon dactylon*, *Artemisia vulgaris*, *Pinus silvestris*, *Plantago lanceolata*, *Holcus lanatus*, *Ostrya virginiana*, *Lolium perenne*, *Poa pratensis*, *Populus nigra*, *Robinia pseudoacacia*, *Pyrethrum spp.*, *Ulmus campestris*, *Parietaria* mix, *Poaceae* mix, *Compositae* mix, *Aceraceae-Fagaceae* mix, and *Betullaceae* mix. Animal allergens included dog epithelium, cat epithelium and feathers mix. Food allergens included wheat flour, barley flour, cornflour, rice flour, soy flour, rye flour, fibers mix and milk proteins. Saline was used as a negative control and histamine chlorhydrate, 10 mg/mL, as a positive control. Since the availability of the different allergens varied during the period of the study depending on the supplier company, not all horses were tested with the same allergens and only 27 allergens were common to every patient. 

**Figure 1:**
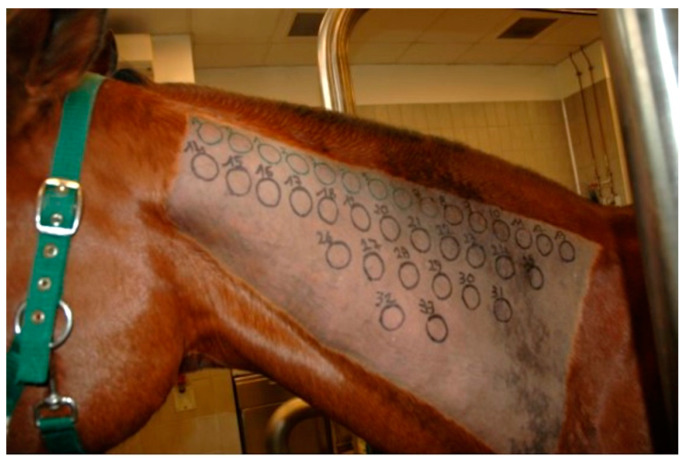
The lateral aspect of the neck has been clipped and circles were drawn with a black marker to indicate injection sites.

The IDT was performed on the lateral aspect of the neck. A rectangular area measuring 20 cm × 40 cm was clipped and cleaned to eliminate gross contamination. A permanent black marker was used to draw circles of 2.5 cm diameter, located 1.5 cm apart from each other, in order to indicate injection sites (Figure 1). A 0.1 mL volume for each allergen, including negative and positive controls, was injected intradermally using a 25-gauge needle. No sedation was necessary for any horse. Injection sites were evaluated 30 min (immediate), 4 h (late-phase), 24 h and 48 h (delayed) after injections, in order to detect different hypersensitivity reaction types. Reactions were judged subjectively by two equally experienced operators through visual inspection and digital palpation and were graded from 0 (no reaction) to 4 (maximum reaction) on the basis of wheal diameter, thickness, turgidity, warmth and achiness [31]. All reactions of grade ≥1 were considered as positive responses.

### 2.3. Statistical Analysis

Horses were divided on the basis of their diagnosis into the SEA and MEA groups. All data were evaluated for normality using a Shapiro–Wilk test. Data for weight were normally distributed, while data for age were normalized by means of logarithmic transformation. The grades of IDT reactions were not normally distributed. Weight and normalized age between groups were compared using an unpaired Student’s *t*-test. The differences in sex distribution between groups were evaluated by means of a Fisher’s exact test. 

A Fisher’s exact test was used to compare the number of horses having positive reactions to any allergen in total and at each evaluation time between groups. The total grades of the reactions, divided into mild-moderate reactions (grades 1–2) or strong reactions (grade 3–4), from any allergen were compared between groups with a Fisher’s exact test. Besides, the number of allergens provoking positive reactions in total and at each evaluation time was compared between groups using a Fisher’s exact test. 

The prevalence of positive reactions and the medians of the grades of the reactions provoked by each specific allergen at every evaluation time were calculated in both groups. The frequency of positive and negative reactions induced by each specific allergen at every evaluation time was compared between groups by means of a Fisher’s exact test. The grades of the reactions provoked by each allergen at every evaluation time were compared between groups using a Mann–Whitney test.

Data are presented as mean ± standard deviation (SD) if normally distributed and as median and interquartile ranges if not normally distributed. Statistical significance was set at *p* < 0.05. Data were analyzed using a commercially available statistical software package (GraphPad Prism 9.1.0 for MacOS; GraphPad Software, San Diego, CA, USA).

## 3. Results

### 3.1. Horses

Thirty-eight horses met the inclusion criteria and were divided into a SEA group (26 horses) and a MEA group (12 horses). The study population consisted of 13 females and 25 males, aged from 2 to 22 years (mean ± SD: 10.79 ± 4.91 years), weighing from 297 to 642 kg (mean ± SD: 498.13 ± 78.61 kg). There was no difference in weight between groups (SEA 512.8 ± 85.79 kg, MEA 466.3 ± 49.35 kg; *p* = 0.09); in contrast, a statistically significant difference was observed for age between groups (SEA 12.77 ± 3.8 years, MEA 6.5 ± 4.34 years; *p* < 0.0001). Even if a greater percentage of males was present in the MEA group (10 males, 2 females) compared to the SEA group (15 males, 11 females), there was no significant difference in sex distribution between groups (*p* = 0.158).

### 3.2. Intradermal Testing

At 30 min, all horses of both groups had a grade three or four reaction to intradermal injection of histamine (positive control); instead, no reaction to saline (negative control) was observed in 19 horses (12 SEA, 7 MEA), while a grade 1 reaction was detected in 19 patients (14 SEA, 5 MEA). 

The frequencies of positive reactions to any allergen and the allergens inducing positive reactions in the SEA and MEA groups at every evaluation time are reported in Table 1. No significant difference was observed in the total number of horses having positive reactions to any allergen between groups. However, at 30 min and 4 h, significantly more horses in the SEA group had positive reactions compared to the MEA group (*p* < 0.0001); in contrast, at 24 h and 48 h, more horses in the MEA group had positive reactions compared to the SEA group (*p* < 0.0001). No significant difference was observed in the total grades of the reactions at any allergen between groups (*p* = 0.563). In total, there was no difference between groups in the number of allergens provoking positive reactions (*p* = 0.348), nor at 4 h (*p* = 0.751) and 48 h (*p* = 0.734). Instead, at 30 min, a significantly higher number of allergens gave positive reactions in the SEA group compared to the MEA group (*p* < 0.0001); at 24 h, more allergens induced positive reactions in the MEA group compared to the SEA group (*p* = 0.0002). 

The frequencies of positive reactions to each allergen at different evaluation times in SEA and MEA horses are reported in Table 2. At 30 min, 36/50 allergens (72%) induced a positive reaction in 100% of the SEA horses, while 28/50 allergens (56%) gave positive responses in 100% of the MEA horses. Reactions of moderate intensity (median grade 2) were induced by 45/50 allergens (90%) in the SEA group, and by 46/50 allergens (92%) in the MEA group. 

At 4 h, 100% of the SEA horses had positive reactions to *Culex spp.*, *Aedes spp.*, *Tabanus spp.* and *Chrysops spp.*, while more than 90% of the SEA horses responded positively to dog epithelium, *Cupressus sempervirens* and *Dermatophagoides* mix. In the MEA group, 100% of the horses had positive reactions to *Aedes*, *Tabanus*, dog epithelium and *Dermatophagoides* mix; more than 90% responded positively to *Parietaria* mix, wheat flour, cornflour, rice flour, soy flour, *Plantago lanceolata*, *Penicillium* mix, *Culex* and *Lepidogliphus destructor*. In the SEA group, strong reactions (median grade 3) were provoked by *Aedes* and dog epithelium, and moderate reactions (median grade 2) were induced by *Tabanus spp.*, *Culex spp.*, *Chrysops spp.* and wheat flour. In the MEA group, strong reactions (median grade 3) were provoked by *Aedes spp.*, *Tabanus spp.* and dog epithelium, and moderate reactions (median grade 2) were induced by *Alternaria alternata*, *Dermatophagoides* mix, *Candida albicans*, *Artemisia vulgaris*, *Plantago lanceolata*, *Robinia pseudoacacia*, wheat flour and soy flour.

At 24 h, *Aedes spp.* induced positive reactions in 100% of the SEA horses, and *Candida albicans* in 81% of the SEA horses; in the MEA group, *Candida albicans* provoked positive responses in 83% and *Tabanus spp.* in 82% of the horses. At 48 h, *Candida albicans* induced positive reactions in 85% of the SEA horses and 92% of the MEA horses. At 24 h and 48 h, *Candida albicans* induced strong reactions (median grade 3) in both the SEA and MEA groups.

No significant differences were observed between groups for the frequency of positive and negative reactions induced by each specific allergen at every evaluation time, with some exceptions: *Alternaria alternata* at 24 h (*p* = 0.022), barley flour at 24 h (*p* = 0.03) and *Penicillium* mix at 4 h (*p* = 0.03) gave significantly more positive reactions in MEA horses compared to the SEA horses, while *Culex spp.* at 30 min (*p* = 0.016) and milk proteins at 48 h (*p* = 0.035) induced significantly more positive reactions in SEA horses compared to the MEA horses. Moreover, no significant differences were observed between groups for the grade of the reactions provoked by each allergen at every evaluation time, except for *Alternaria alternata* at 4 h (*p* = 0.014) and 24 h (*p* = 0.017), *Candida albicans* at 4 h (*p* = 0.04), corn flour at 30 min (*p* = 0.021) and 4 h (*p* = 0.046), which provoked more intense reactions in the MEA group compared to the SEA group, and milk proteins at 48 h (*p* = 0.035), which induced stronger reactions in the SEA group compared to the MEA group. 

## 4. Discussion

To the authors’ knowledge, this is the first study comparing results of intradermal testing between horses affected by severe and mild-moderate equine asthma, even though no healthy horses were included in the present study. The SEA and MEA groups were weight- and sex-matched; however, the MEA horses were significantly younger compared to the SEA horses. In fact, SEA usually affects adult horses over seven years of age, while MEA can affect horses of any age, but it is more commonly reported in young horses [7].

SEA horses showed a higher probability to develop positive reactions to an allergen at 30 min and 4 h, compared to the MEA horses; this result is in accordance with a previous study, in which SEA horses developed more positive reactions at 30 min and 4 h, compared to healthy horses [10]. This suggests that a type-I hypersensitivity response may be involved in the pathogenesis of the SEA, based on IgE-mediated mast cell degranulation. Interestingly, in our study, at 24 and 48 h, the MEA horses had more positive reactions compared to the SEA horses: this could suggest that delayed type-IV hypersensitivity could be more implicated in the pathogenesis of mild forms of EA compared to more severe phenotypes. This hypothesis is further supported by the fact that the SEA horses had positive reactions to a greater number of allergens at 30 min, compared to the MEA horses, while MEA horses responded to more allergens at 24 h compared to the SEA horses. 

However, in total time, there was no difference nor in the frequency of positivity nor in the number of allergens provoking positive reactions between the SEA and MEA horses. Previous studies reported that SEA horses develop overall more positive reactions compared to healthy horses [12,27] and respond to a greater number of allergens [26,27]. In contrast, in one study, more positive reactions were observed among horses without atopy, compared to the SEA horses: however, most environmental allergens used in that investigation included grasses, weeds, trees and insects, which are mostly present outdoors, where non-atopic horses had been kept for prolonged periods; therefore, non-atopic horses had been previously more exposed and sensitized to those environmental allergens compared to the SEA group [31]. The authors of another study comparing different allergy tests in asthmatic horses observed no significant difference between the IDT results of the healthy and the asthmatic group: however, it included a small number of patients, and only type I hypersensitivity responses were evaluated [24]. In our study, no difference in the grades of the reactions was observed between the SEA and MEA groups; previous studies showed that SEA horses were likely to develop not only a greater number of reactions, but also of a stronger intensity compared to healthy horses, suggesting that asthmatic horses have an increased sensitivity to specific antigens [26,28]. 

In the present study, at 30 min, all the SEA horses responded to 72% of the allergens, while all the MEA horses responded to 56% of the allergens with a moderate intensity grade, suggesting that asthmatic horses have a tendency to develop immediate hypersensitivity responses to most common allergens. However, an evaluation of the IDT reactions at 30 min only allows to identify the tendency of the patient to overreact to common allergens, without providing useful information about the specific antigens that could be considered as triggers of the allergic response. An evaluation of the response at 4 h seems to be more useful in identifying specific allergens in SEA horses. In this experiment it was found to correspond to the late-phase of type I hypersensitivity, which is likely involved in the pathogenesis of allergic reactions, and is more allergen-selective compared to the immediate type (30 min). 

Previous studies also suggested that the observation time point at 4 h is the most significative when performing an IDT [27,31], with optimal sensitivity and specificity [27]. In our study, positive reactions at 24 h significantly decreased in the SEA group, suggesting less involvement of type-IV hypersensitivity, compared to type I; however, in the MEA group, positivities at 24 h were slightly more than those at 4 h, indicating that delayed response could be implicated in mild forms of asthma and, therefore, this evaluation time should be taken into consideration for the identification of the most allergenic antigens in MEA horses. A previous study suggested that observation of IDT responses at 24 h is pivotal in SEA horses, since some positive delayed reactions could not be preceded by a type I response, even if it rarely occurs [26]. The present study is the first one evaluating IDT reactions also at 48 h; however, positive reactions were rarely observed and the only allergen provoking intense responses was *Candida albicans* in both groups. 

Nevertheless, the role of *Candida albicans* in the IDT is controversial: in fact, this allergen commonly induces delayed reactions also in healthy non-atopic horses [31] and, in human medicine, it has been used to evaluate the overall capacity of a subject to develop delayed hypersensitivity responses [36]. Therefore, positive reactions to *Candida albicans* at 24 or 48 h should be considered of scarce clinical significance; *Candida albicans* could rather be used as a positive control for delayed type-IV hypersensitivity reactions, as well as histamine is utilized for immediate responses. In conclusion, it is reasonable that a complete evaluation of IDT results should include observation of the responses at 30 min, 4 h and 24 h: a reading at 30 min allows to evaluate whether the patient is hyperreactive to common allergens. At 4 h, it is possible to identify specific allergens responsible for type-I hypersensitivity reactions; at 24 h, eventual delayed responses (which may or may not be preceded by an immediate or late-phase reaction) could be observed. Finally, an evaluation at 48 h in the present study did not provide any adjunctive relevant information.

Among our patients, at 4 h, 100% of the SEA horses developed positive reactions to *Culex spp.*, *Aedes spp.*, *Tabanus spp.* and *Chrysops spp.*, suggesting that hypersensitivity to the class of allergens “insects” could be common among asthmatic horses; also 100% of the MEA horses reacted to *Aedes spp.* and *Tabanus spp.*, while more than 90% responded to *Culex spp. Aedes spp.* induced strong reactions in both the SEA and MEA groups, and also provoked delayed reactions in 100% of the SEA horses at 24 h; this insect, commonly known as “tiger mosquito”, has spread worldwide during the past few decades [37] and our results suggest that its role as an allergen deserves to be taken much into consideration among EA patients. 

*Tabanus spp.* provoked moderate-intensity responses in the SEA group and strong reactions in the MEA group; however, the role of this allergen in IDT has been questioned since it seems to induce too many positive reactions also among healthy non-atopic horses [27,32]. Interestingly, a correlation between insect bite hypersensitivity (IBH) and airways hyperreactivity has been recently demonstrated [38]: in fact, horses affected by IBH seem to have a higher risk of being concurrently asthmatic, and vice versa [39]. This suggests that multiple hypersensitivities could share a common immunogenetic background or route of sensitization; concurrent different hypersensitivity manifestations are also observed among human patients affected by asthma and atopic dermatitis [40], dogs with allergic dermatitis, conjunctivitis and rhinitis [41], and cats with feline asthma and pruritic dermatosis [42]. Considering the high allergenic potential that insect antigens seem to have in horses, protecting them from insect bites becomes pivotal in the control of allergic manifestations, including asthma.

In addition to insects, the present study identified other relevant allergens which induced moderate-strong reactions in many of our patients of both the SEA and MEA groups, including dog epithelium and *Dermatophagoides* mix. In addition, some pollens frequently provoked positive reactions: in the SEA group *Cupressus sempervirens*, while in the MEA group *Parietaria* mix, *Artemisia vulgaris*, *Robinia pseudoacacia* and *Plantago lanceolata*. In MEA horses, a high hypersensitivity to different food flours was detected too. Previous investigations showed a high allergenicity of *Aspergillus fumigatus*, *Alternaria alternata*, *Tyrophagus putrescentiae*, *Micropolyspora faeni*, *Saccharopolyspora rectivirgula*, *Aspergillus terreus*, *Eurotium amstelodami*, *Geotrichum candidum*, *Wallemia sebi* and *Dermatophagoides spp.* in asthmatic horses [11,26,32,43,44,45,46,47,48,49]. 

The present study confirms a great reactivity to *Dermatophagoides spp.*, formerly reported as one of the allergens inducing the strongest reactions [32] in both the SEA and MEA horses. Moreover, a moderate hypersensitivity to *Alternaria alternata* was shown in our study among MEA horses: this allergen has atmospheric peaks during the seasons in which the majority of exacerbations of SEA occur and, in a study about skin test using 16 relevant aeroallergens, it induced the largest wheal diameter in SEA horses [28]. In human medicine, it is regarded as one of the most clinically important moulds worldwide, playing a major role in the pathogenesis of allergic asthma: sensitization against *Alternaria alternata* is considered an important risk factor for greater asthma severity and acute asthma exacerbations of affected patients [50,51,52]. 

The allergenic role of *Aspergillus fumigatus* and *Tyrophagus putrescentiae* has been questioned; in fact, in different studies, they have shown to induce positive reactions also in healthy horses [26,28,30]. However, in our study they did not appear to be particularly allergenic in the SEA or the MEA horses. The different results observed between our study and previous analogous investigations could be explained by the fact that they were performed in different geographic areas, characterized by different climates and concentrations of airborne allergens. Moreover, to date, the studies about IDT in SEA horses lack standardization and IDT reactions are generally subjectively evaluated: some studies proposed to measure the diameter of the wheals and to establish cut-off values to distinguish positive and negative results [27,28,32]. Though the warmth of the wheals could be estimated by means of thermography, the assessment of turgidity and achiness relies on the operator’s sensitivity and cannot be objectively measured. The lack of objective measurements represents one of the main limitations of IDT. Furthermore, in our study, all IDT responses of grade ≥1 were considered as positive reactions, while some previous studies only considered reactions of grade ≥1.5 [53] or even ≥3 [27]. In the present work, it is reasonable that subclinical hypersensitivities were also included; therefore, the evaluation of the intensity of the reactions by assignment of a score was essential in order to interpret positive results.

Since positivity is not specific for asthma but only identifies atopic status, confirmation of the clinical significance of the identified allergens in the pathogenesis of asthma should be pursued. To this aim, allergen avoidance is indicated [54] to observe the eventual remission of symptoms and prevention of exacerbations. However, avoidance strategies are not always feasible, since some aeroallergens are ubiquitous—in this case, the most accurate evaluation of the diagnostic value of IDT may be to compare these tests with the gold standard of induction of clinical disease by a provocative inhalation challenge using the identified allergens, which would provide more substantial evidence of their role as causative antigens [26,27]. 

When the confirmation of the pathogenic role of specific allergens is obtained, selected patients may be treated with ASIT [26], which is defined as the administration of gradually increasing quantities of an allergen extract to an allergic subject to ameliorate the symptoms associated with subsequent exposure to the causative allergen [55]. Today, immunotherapy is, according to systematic reviews and meta-analyses, an accepted treatment for human allergic asthma, helpful in alleviating symptoms and reducing the needing for medication [56,57]. There is also some evidence for the successful treatment of feline asthma with immunotherapy, but studies to date are limited to experimentally sensitized cats [58]. In equine medicine, there are only few studies about immunotherapy for the treatment of asthma [59], which were not controlled and included only small numbers of horses; therefore, it has not been widely used to date [60]. However, based on the similarities with human asthma [1,2], it is reasonable that ASIT could also be effective for the treatment of EA and future investigations should be addressed to verify this hypothesis.

## 5. Conclusions

In conclusion, mostly type-I hypersensitivity response seems to be involved in the pathogenesis of SEA, and the evaluation of IDT responses at 4 h is essential for the identification of specific antigens responsible for allergic reactions. Insects, *Dermatophagoides spp.* and dog epithelium, when induced in the MEA and SEA horses of this study, provided the most significant hypersensitivity responses and could therefore be considered as the main allergenic antigens in our geographic area. Future investigations are required to evaluate clinical significance of IDT and the efficacy of ASIT for the treatment of EA patients.

## Figures and Tables

**Table 1:** Frequencies of positive reactions to any allergens and of the allergens inducing positive reactions at each evaluation time in the severe equine asthma (SEA) and mild-moderate equine asthma (MEA) groups.

	Total Time	30 min	4 h	24 h	48 h
Positivities SEA	48.98%	98.34%	76.63%	15.09%	5.84%
Positivities MEA	50%	84.12%	47.64%	49.34%	18.90%
	ns	*p* < 0001	*p* < 0.0001	*p* < 0.0001	*p* < 0.0001
Allergens SEA	49.05%	98.73%	76.73%	15%	5.74%
Allergens MEA	50.33%	93.95%	77.50%	22.91%	6.24%
	ns	*p* < 0.0001	ns	*p* = 0.0002	ns

**Table 2:** Frequencies of positive reactions to each allergen at different evaluation times in the SEA and MEA groups.

ALLERGEN	30 min		4 h		24 h		48 h	
	SEA	MEA	SEA	MEA	SEA	MEA	SEA	MEA
*Acarus siro*	92%	100%	81%	75%	8%	0%	4%	0%
*Aceraceae-Facaceae* mix	96%	92%	65%	58%	0%	8%	0%	0%
*Aedes spp.*	67%	50%	100%	100%	100%	67%	33%	17%
*Alternaria alternata*	100%	100%	69%	83%	8%	42%	4%	17%
*Artemisia vulgaris*	100%	92%	85%	75%	12%	33%	0%	0%
*Aspergillus fumigatus*	100%	92%	81%	67%	12%	17%	4%	0%
Barley flour	100%	92%	80%	83%	4%	33%	4%	0%
*Betullaceae* mix	96%	92%	77%	67%	8%	8%	0%	0%
*Candida albicans*	96%	92%	85%	83%	81%	83%	85%	92%
Cat epithelium	100%	100%	85%	67%	8%	17%	4%	8%
*Chrysops spp.*	100%	100%	100%	60%	36%	0%	21%	0%
*Cladosporium spp.*	100%	92%	73%	75%	4%	8%	4%	0%
*Compositae* mix	100%	100%	88%	82%	17%	27%	0%	9%
Corn flour	100%	100%	65%	92%	15%	8%	4%	0%
*Corylus avellana*	100%	100%	65%	75%	4%	8%	0%	0%
*Culex spp.*	100%	64%	100%	91%	35%	45%	0%	9%
*Culicoides spp.*	100%	82%	82%	73%	29%	18%	6%	18%
*Cupressus sempervirens*	100%	92%	92%	67%	12%	8%	0%	0%
*Cynodon dactylon*	96%	92%	69%	67%	8%	0%	0%	0%
*Dermatophagoides* mix	100%	100%	92%	100%	19%	42%	4%	0%
Dog epithelium	100%	100%	96%	100%	31%	50%	19%	25%
Feathers mix	100%	100%	67%	43%	33%	14%	0%	0%
Fibers mix	100%	100%	67%	57%	10%	0%	0%	0%
*Gliciphagus domesticus*	100%	90%	65%	60%	4%	10%	0%	0%
*Helmintosporium sativum*	96%	100%	73%	83%	8%	25%	0%	0%
*Holcus lanatus*	100%	100%	65%	75%	10%	8%	0%	0%
*Lepidogliphus destructor*	100%	90%	80%	90%	15%	20%	4%	0%
*Lolium perenne*	100%	100%	73%	75%	60%	25%	0%	8%
Milk proteins	100%	100%	60%	75%	33%	25%	0%	0%
*Musca domestica*	67%	50%	67%	33%	8%	0%	60%	0%
Mycophytes mix	100%	100%	79%	82%	0%	27%	0%	0%
*Olea europea*	96%	92%	77%	67%	4%	8%	0%	0%
*Ostrya virginiana*	96%	92%	65%	67%	19%	17%	0%	0%
*Parietaria* mix	100%	100%	85%	92%	15%	17%	0%	17%
*Penicillium* mix	96%	100%	54%	92%	8%	25%	4%	8%
*Pinus silvestris*	96%	100%	67%	82%	17%	27%	0%	0%
*Plantago lanceolata*	100%	100%	73%	92%	19%	25%	4%	0%
*Poa pratensis*	100%	100%	58%	67%	8%	17%	0%	8%
*Poaceae* mix	96%	100%	81%	75%	4%	17%	0%	0%
*Populus nigra*	100%	100%	80%	88%	20%	25%	20%	0%
*Pyrethrum spp.*	100%	86%	67%	71%	33%	0%	0%	0%
Rice flour	100%	100%	88%	92%	12%	42%	0%	8%
*Robinia pseudoacacia*	100%	88%	60%	75%	20%	0%	0%	0%
Rye flour	100%	88%	60%	75%	60%	13%	40%	13%
Soy flour	100%	100%	85%	92%	12%	25%	4%	8%
*Tabanus spp.*	100%	73%	100%	100%	47%	82%	29%	27%
Trichoepidermophytes mix	88%	100%	75%	70%	10%	10%	5%	0%
*Tyrophagus putrescentiae*	100%	91%	75%	73%	17%	18%	8%	0%
*Ulmus campestris*	100%	100%	44%	25%	11%	0%	0%	0%
Wheat flour	100%	100%	77%	92%	15%	25%	0%	0%

## Data Availability

The data presented in this study are available on request from the corresponding author.
